# Heterologous Expression of a Thermostable α-Glucosidase from *Geobacillus* sp. Strain HTA-462 by *Escherichia coli* and Its Potential Application for Isomaltose–Oligosaccharide Synthesis

**DOI:** 10.3390/molecules24071413

**Published:** 2019-04-10

**Authors:** Fan Zhang, Weiyang Wang, Fatoumata Binta Maci Bah, Chengcheng Song, Yifa Zhou, Li Ji, Ye Yuan

**Affiliations:** Jilin Province Key Laboratory on Chemistry and Biology of Changbai Mountain Natural Drugs, School of Life Sciences, Northeast Normal University, Changchun 130024, China; zhangf508@nenu.edu.cn (F.Z.); wangwy576@nenu.edu.cn (W.W.); mariamatimbi@yahoo.com (F.B.M.B.); songcc225@nenu.edu.cn (C.S.); zhouyf383@nenu.edu.cn (Y.Z.)

**Keywords:** α-glucosidase, isomaltose-oligosaccharides, heterologous expression, transglycosylation, thermostability

## Abstract

Isomaltose–oligosaccharides (IMOs), as food ingredients with prebiotic functionality, can be prepared via enzymatic synthesis using α-glucosidase. In the present study, the α-glucosidase (GSJ) from *Geobacillus* sp. strain HTA-462 was cloned and expressed in *Escherichia coli* BL21 (DE3). Recombinant GSJ was purified and biochemically characterized. The optimum temperature condition of the recombinant enzyme was 65 °C, and the half-life was 84 h at 60 °C, whereas the enzyme was active over the range of pH 6.0–10.0 with maximal activity at pH 7.0. The α-glucosidase activity in shake flasks reached 107.9 U/mL and using 4-Nitrophenyl β-D-glucopyranoside (*p*NPG) as substrate, the *K*_m_ and *Vmax* values were 2.321 mM and 306.3 U/mg, respectively. The divalent ions Mn^2+^ and Ca^2+^ could improve GSJ activity by 32.1% and 13.8%. Moreover, the hydrolysis ability of recombinant α-glucosidase was almost the same as that of the commercial α-glucosidase (*Bacillus stearothermophilus*). In terms of the transglycosylation reaction, with 30% maltose syrup under the condition of 60 °C and pH 7.0, IMOs were synthesized with a conversion rate of 37%. These studies lay the basis for the industrial application of recombinant α-glucosidase.

## 1. Introduction

Isomalto–oligosaccharides (IMOs) are a group of functional oligosaccharides with a degree of polymerization (DP) ranging 2–10, which consist of glucosyl saccharide units linked by α-1, 6-glycosidic linkages [1]. Isomaltose, panose, isomaltotriose and tetrasaccharides are defined as the main functional components of IMOs [2]. As functional oligosaccharides, which are non-digestible in nature, IMOs have the function of regulating intestinal flora by promoting the proliferation of *Bifidobacterium* and *Lactobacillus* in the human intestinal tract [3], and also have the additional properties of: low caloric value [4], the promotion of gastrointestinal peristalsis, and the improvement of constipation and lipid metabolism [5,6]. Based on these functions, IMOs are widely utilized as prebiotics, food additives and feed ingredients [7,8,9,10].

IMOs are commonly produced from starch via enzymatic conversion using α-amylase, β-amylase, pullulanase and transglucosidase [11]. The typical process for IMO production usually comprises three steps: (1) liquefaction, where starch is liquefied by a thermostable α-amylase to produce dextrin; (2) saccharification, where the dextrin is then saccharified by β-amylase and pullulanase; (3) transglycosylation, where α-glucosidase is used for converting maltose (saccharified products) into IMOs [12]. Since commercial α-amylase, β-amylase and pullulanase are well-studied and their industrial uses are relatively mature, studying the transglycosylation reaction catalyzed by α-glucosidase is the key step in IMO production. α-glucosidase (EC 3.2.1.20, GH13) catalyzes the liberation of glucose from non-reducing terminals of substrates [13]. In addition to the hydrolysis activity, some α-glucosidases, for instance, those from *Aspergillus niger* [14], *Bacillus stearothermophilus* [15] and *Xantophyllomyces* *dendrorhous* [16], possess transglycosylation activity, which can transfer a glucosyl residue to the 6-OH group of the non-reducing glucose unit and yield IMOs [17].

To date, a large number of α-glucosidases with transglycosylation activity have been identified and characterized. *Aspergillus niger* α-glucosidase was reported to have high transglycosylation activity, and was hence successfully applied to produce IMOs [18,19]. With the increasing commercial interest and industrial application prospects, α-glucosidase production has drawn extensive attention. Generally, α-glucosidase from *A. niger* is stable below 50 °C, restricting the current commercial transglycosylation reaction optimally operated at around 50 °C. However, if the temperature of the transglycosylation reaction can be increased, the faster reaction rate, higher conversion rate and decreased viscosity of the substrate would be advantageously achieved. Previously, α-glucosidase from *Geobacillus* sp. strain HTA-462 (an isolate from Mariana Trench sediment) was characterized, and the results showed that the wild-type enzyme exhibited good thermostability and good transglycosylation activity [20], indicating that GSJ has potential application in the IMO industry.

In the present study, for the first time, *E. coli* BL21 (DE3) was selected as the host strain for the expression of the gene encoding α-glucosidase (GSJ) from *Geobacillus* sp. strain HTA-462. The ability of the purified recombinant α-glucosidase to synthesize IMOs was investigated and our studies aimed to provide a platform for the development of GSJ into an industrial enzyme.

## 2. Results

### 2.1. Expression and Purification of α-Glucosidase

**Figure 1:**
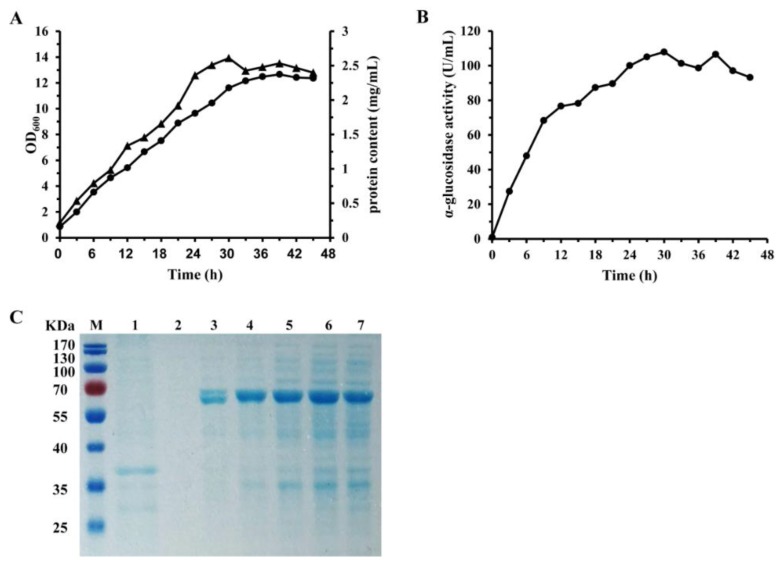
Recombinant α-glucosidase (GSJ) production from *E. coli* BL21 (DE3). (**A**) Time profiles for batch cultivations of recombinant *E. coli* in shake flasks: ●, OD600 of the bacteria cells; ▲, the protein concentration of recombinant GSJ. (**B**) The intracellular enzyme activity of recombinant GSJ. (**C**) Sodium dodecyl sulfate-polyacrylamide gel electrophoresis (SDS-PAGE) analysis of recombinant GSJ. Samples were run on a 10% SDS-PAGE gel. Proteins were visualized using Coomassie Brilliant Blue G-250. Lane M, molecular weight markers; Lane 1, *E. coli* BL21-pET28a; Lane 2-7, intracellular supernatant of recombinant *E. coli* after isopropyl β-D-1-thiogalactopyranoside (IPTG) induction at 0 h, 6 h, 12 h, 21 h, 27 h and 33 h.

The prokaryotic expression system (*E. coli* BL21 (DE3)/pET28a-gsj) was established for the inducible expression of GSJ. The synthesized gene was sub-cloned into the *EcoR* I and *Xho* I sites of pET-28a (+). The transformation of the designated pET28a/gsj into *E. coli* BL21 (DE3) yielded the recombinant strain. Since we used the T7 promoter-based expression system in this study, isopropyl β-D-1-thiogalactopyranoside (IPTG) was used for the induction of protein overexpression. The expression efficiency of the engineered *E. coli* was explored in a shake flask. Under the optimized culture conditions, the intracellular α-glucosidase activity kept rising rapidly and reached a maximum value of 107.9 U/mL at 30 h (Figure 1).

**Figure 2:**
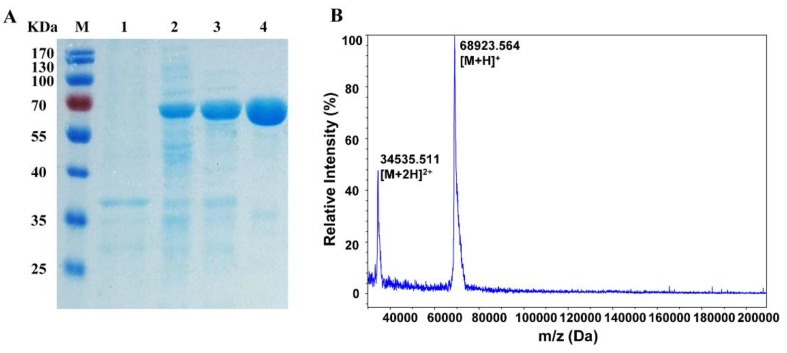
(**A**) The purification of recombinant GSJ from *E. coli* BL21 (DE3). Lane M, molecular weight markers; Lane 1, *E. coli* BL21-pET28a; Lane 2, crude enzyme; Lane 3, heat treatment; Lane 4, purified GSJ. (**B**) MALDI-TOF analyses of recombinant GSJ. The major signals are labelled as [M + 2H]^2+^ and [M + H]^+^, respectively.

The enzyme purification process is summarized in Table 1. The N-terminal his-tagged recombinant α-glucosidase from the cell-free extract was purified through a combination of heat treatment and chromatographic separation. GSJ was purified 6.6-fold, with a specific activity of 283.3 U/mg and an overall recovery of 73.7%. Sodium dodecyl sulfate-polyacrylamide gel electrophoresis (SDS-PAGE) analysis showed that the purified recombinant enzyme was detected as a single protein band with an apparent molecular mass of about 70 kDa, and the exact molecular weight, determined using MALDI-TOF mass spectrometry, was 68.9 KDa, matching the theoretically calculated value (Figure 2B).

### 2.2. Characterization of the Recombinant Enzymes

#### 2.2.1. Temperature Optimum and Thermostability

**Figure 3:**
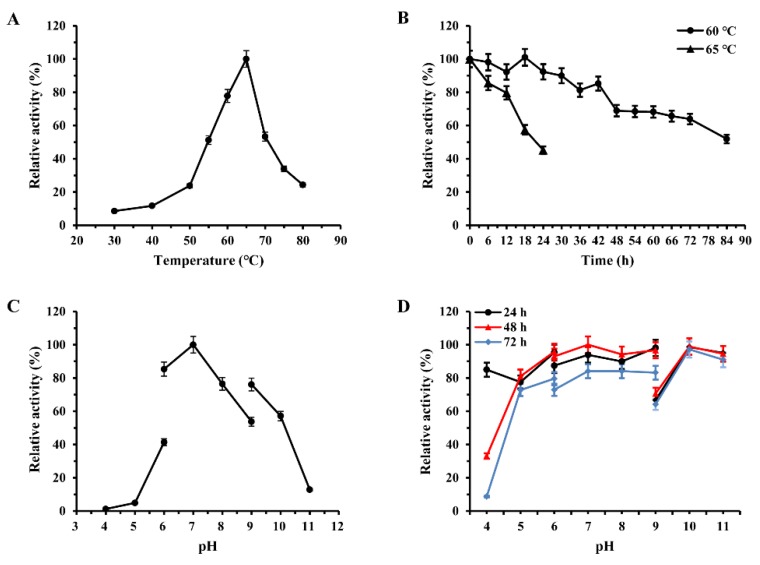
Effect of temperature and pH on the activity and stability of recombinant GSJ. (**A**) Optimum temperature. Activity was measured between 30 °C and 90 °C at pH 7.0 for 10 min. The activity at optimum temperature was defined as 100%. (**B**) Thermostability. Assays were carried out in 50 mM phosphate buffer (pH 7.0) at 65 °C for 10 min after the incubation of the enzyme at 60 °C (●) or 65 °C (▲). The activity without heat treatment was defined as 100%. (**C**) Optimum pH. Assays were carried out at 65 °C for 10 min in buffers ranging in pH from 4.0 to 11.0. The activity at optimum pH was defined as 100%. (**D**) pH stability. Assays were carried out after the incubation of the enzyme in buffers ranging from pH 4.0–11.0 at 4 °C for 24–72 h.

The optimum temperature curve of the purified enzyme indicated that the activity increased with temperature until it peaked at 65 °C, and the enzyme retained above 50% activity between 55 °C and 75 °C (Figure 3A). The thermostability of the recombinant enzyme was determined at pH 7.0 and temperatures of 60 °C and 65 °C. As shown in Figure 3C, the enzyme still retained 90% of activity after 30 h at 60 °C, and with a half-time of 84 h. Upon incubation at 65 °C, the enzyme showed 57% residual activity after 18 h. A GSJ with good thermostability and high optimum temperature is beneficial for applications operating at a high temperature for a long time in industry.

#### 2.2.2. pH Optimum and Stability

The enzyme exhibited high enzymatic activity (>75%) between pH 6.0 and 9.0, and the optimum pH was 7.0 (Figure 3B). Furthermore, GSJ was stable at a wide range of pH values, of 6.0–10.0. Additionally, the GSJ retained 93.9% and 84.1% after incubation for 48 h and 72 h at pH 7.0, respectively. It also retained almost initial activity after incubation for 72 h at pH 10.0 (Figure 3D). 

#### 2.2.3. Kinetic Studies

**Figure 4:**
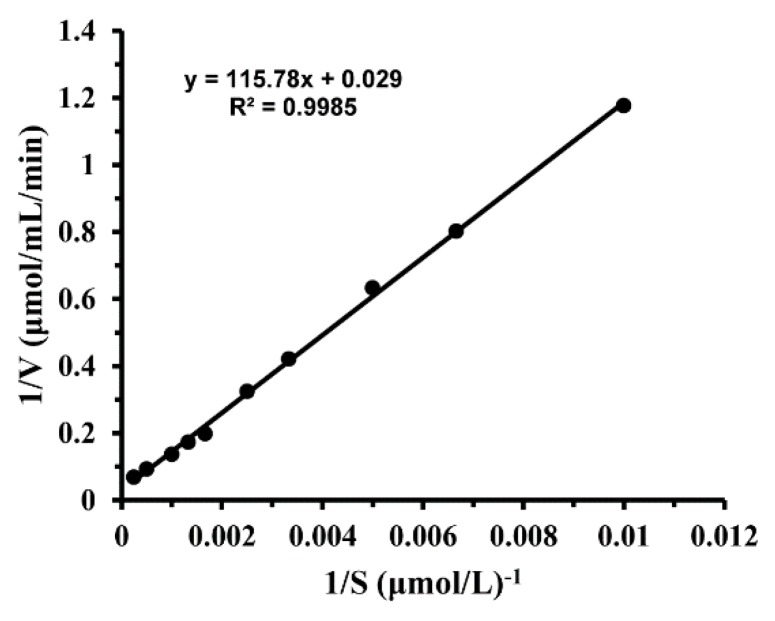
Lineweaver–Burk plot of recombinant GSJ.

The kinetic parameter of the enzyme was analyzed using 4-Nitrophenyl β-D-glucopyranoside (*p*NPG) as the substrate at 65 °C and pH 7.0 (Figure 4). As shown in Table 2, the kinetic constant *K*m was found to be 2.32 mM and the catalytic efficiency *k*_cat_/k_m_ was 151.6 mM^−1^s^−1^.

#### 2.2.4. Metal Requirements 

**Figure 5:**
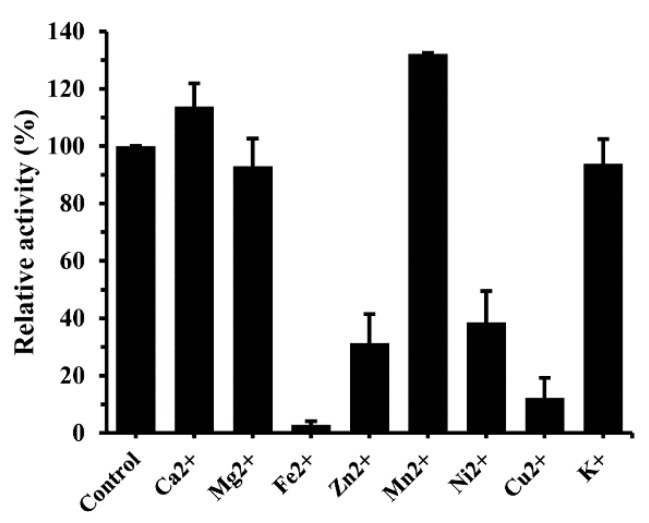
Effect of metal ions on recombinant GSJ. The activity was assayed after the incubation of the enzyme (30 ng) in 50 mM phosphate buffer (pH 7.0) containing 1 mM metal ion at 65 °C for 1 h.

Previously, Mn^2+^ was reported to stimulate the activity of α-glucosidase from *Thermotoga maritima* [21], Ca^2+^ was required for the catalytic efficiency of the enzyme from *E. coli* [22], and *Mucor racemosus* α-glucosidase was able to be stimulated by Al^3+^, Ca^2+^, Mg^2+^ and Na^+^ [23]. In this study, the effect of metal ions on the activity of GSJ was analyzed by preincubating the enzyme with 1 mM metal ions at 65 °C for 1 h. As shown in Figure 5, Mg^2+^ and K^+^ did not affect the enzyme activity of GSJ, Cu^2+^, Zn^2+^ and Ni^2+^ strongly inhibited the enzyme activity by 12.3%–38.6%, while Fe^2+^ almost completely inhibited the enzyme activity. However, its activity was significantly improved by 113.8% and 132.1% in the presence of Ca^2+^ and Mn^2+^.

#### 2.2.5. Substrate Specificity

Nine *p*NP derivatives were chosen as substrates to determine the substrate specificity of GSJ. As the results show in Table 3, GSJ exhibited high activity and specificity toward *p*NPαGlu, whereas the enzyme showed no activity against the other eight *p*NP derivatives (*p*NPβGlu, *p*NPαAra*p*, *p*NPαGal, *p*NPβGal, *p*NPαAra, *p*NPαRha, *p*NPβMan, *p*NPβXyl). The enzyme exclusively hydrolyzed α-1, 4-linked terminal glucose residue.

### 2.3. Hydrolysis Activity

**Figure 6:**
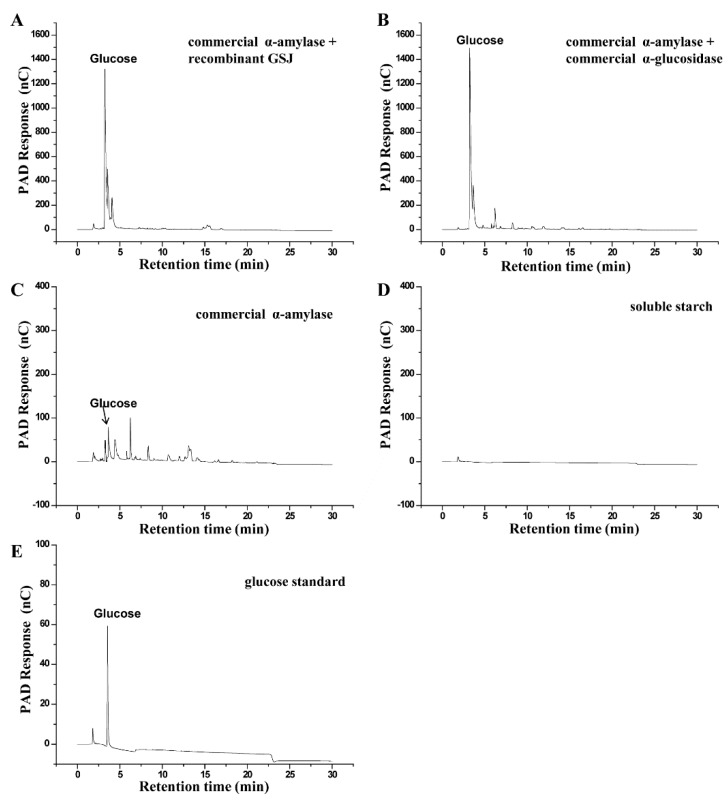
Identification of the hydrolysis products by HPAEC-PAD-200. The hydrolysis products of soluble starch by commercial α-amylase and recombinant GSJ (**A**), commercial α-amylase and commercial α-glucosidase (**B**), commercial α-amylase (**C**). (**D**) Soluble starch. (**E**). Standard samples of glucose.

α-glucosidase is a typical exo-type glycosidase, which could cleave the terminal α-1, 4-glucosidic linkage from the non-reducing ends of oligosaccharides to form glucose. To determine the hydrolysis activity of the recombinant GSJ, commercial α-amylase was utilized to degrade soluble starch to oligosaccharides, which was subsequently hydrolyzed to glucose by the recombinant GSJ/commercial α-glucosidase (*Bacillus. Stearothermophilus*, E-TSAGS, Megazyme, Ireland), and the degradation products were analyzed by high-performance anion-exchange chromatography (HPAEC). When using α-amylase alone, the degradation products were mainly oligosaccharides and a small amount of glucose. However, when α-amylase and GSJ/commercial α-glucosidase were combined, the degradation products were almost entirely glucose (Figure 6), suggesting that the recombinant enzyme has high hydrolysis activity, the same as the commercial enzyme.

### 2.4. Transglycosylation Activity of Recombinant α-Glucosidase

**Figure 7:**
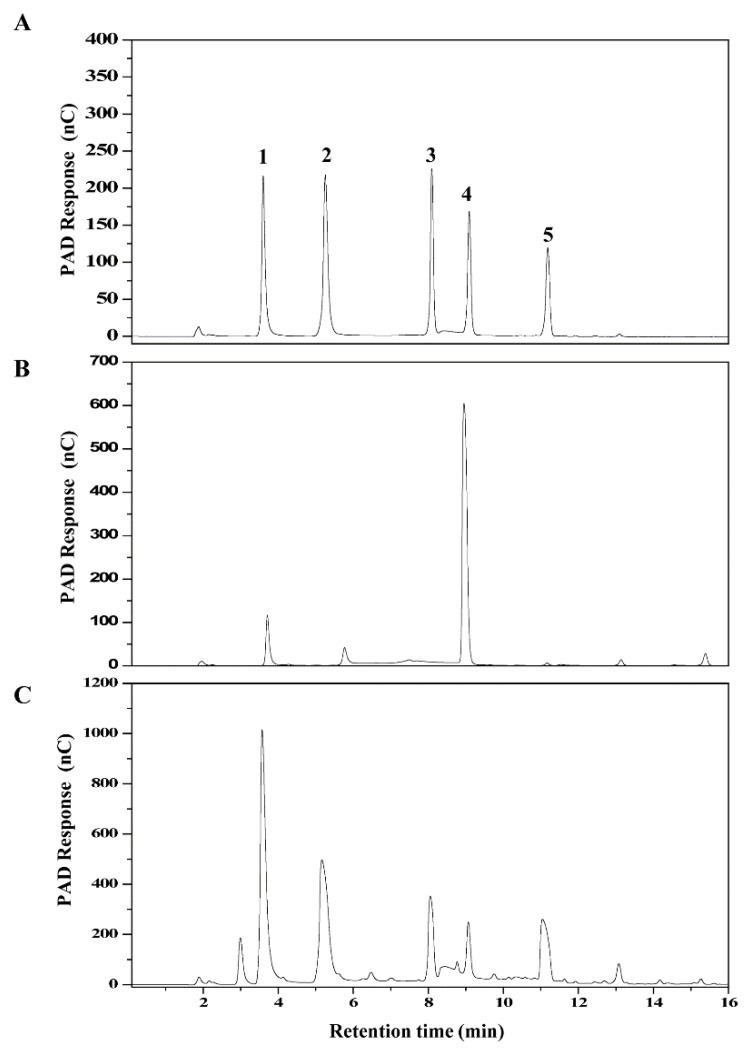
Identification of the transglycosylation products by HPAEC-PAD-200. (**A**) Standard samples of glucose (1), isomaltose (2), isomaltotriose (3), maltose (4) and panose (5). (**B**) maltose substrate (control group). (**C**) Transglycosylation products by recombinant GSJ.

To determine the transglycosylation activity of GSJ, the recombinant α-glucosidase was incubated with 30% (w/v) maltose solution at 60 °C. The progress of the reaction was monitored by HPAEC, and IMO standards were used to identify the retention time of the components. As shown in Figure 7, after 2 h of the reaction, more than 80% of the maltose was consumed, and glucose and a series of transglycosylation products (isomaltose, panose and isomaltotriose) were formed and clearly assigned by HPAEC. Isomaltose, panose and isomaltotriose were measured for total IMOs, and IMOs were synthesized with approximately 37% conversion in 2 h by the recombinant enzyme.

## 3. Discussion

Due to the transglycosylation activity, α-glucosidase is utilized in IMO production [24]. α-amylase, β-amylase and pullulanase hydrolyses starch to maltose, which subsequently transglycosylates to IMOs by α-glucosidase. Currently, *A. niger* α-glucosidase has been applied to produce IMOs in industry, and the relatively mature technology is used by Amano Enzymes Inc., Japan [19]. IMOs, one of the emerging prebiotics, have proved their beneficial effects on bifidogenic flora, bowel function and metabolism, as well as on the immune system [17]. In response to consumers paying more attention to personal health and well-being and increasing demand, the production of IMOs has been promoted in recent years. In order to enhance the IMO yield, many technologies have been developed, such as heterologous expression and the immobilization of α-glucosidase, the combination of different enzymes, and enzymes being bound to living cells. Recombinant enzyme technology was reported to significantly improve IMO production [12].

Thermostable enzymes, which have the advantage of high reactivity, better yield, high stability, low viscosity and little contamination, are important and highly attractive candidates for industrial applications [25]. Previously, α-glucosidase from the *Geobacillus* sp. strain HTA-462 was identified and characterized, and the encoding gene was sequenced and expressed in *Bacillus subtilis* ISW1214 [20]. *E. coli* is the preferred host for recombinant protein expression due to its high-level production of heterologous proteins, fast growth and simple culture conditions. Therefore, in order to improve the enzyme activity and study of the production of IMOs by GSJ, in the present study the gene encoding α-glucosidase from *Geobacillus* sp. strain HTA-462 was cloned and expressed in *E. coli* BL21 (DE3). The enzyme activity of the recombinant GSJ in shake flasks reached 107.9 U/mL. However, the biochemical properties of the GSJ expressed in *E. coli* revealed dissimilarity from the wild-type enzyme. The optimum temperature of the wild-type GSJ was 60 °C, while, in contrast, the recombinant GSJ showed a shift in optimum temperature to 65 °C. The optimal pH of the wild-type GSJ was 9.0 in the glycine-NaOH buffer, while the optimum pH of the recombinant GSJ was 7.0 in phosphate buffer, and it maintained more than 70% activity ranging from pH 6.0 to 10.0. Significantly, recombinant GSJ exhibited higher thermostability. At 60 °C, the half-life of the recombinant GSJ was 84 h, which was 6.3-fold greater than that of the wild-type. The big difference in thermostability was probably caused by the detection at different pHs and in different buffer systems. Notably, in comparison to the recombinant α-glucosidase from *A. niger*, the recombinant GSJ exhibited greater thermostability with a longer half-life, which was 28-fold greater than that of the *A. niger* α-glucosidase (half-life of 3.0 h at 60 °C) [18]. The good thermal stability suggests that GSJ has potential for application in IMO industries. 

Both the hydrolysis and transglycosylation activities of the recombinant GSJ were determined. α-amylase and the recombinant α-glucosidase/commercial α-glucosidase were simultaneously used to convert soluble starch. The results showed that both the α-glucosidase and the commercial α-glucosidase could almost completely hydrolyze the soluble starch to glucose in only 1 h, indicating that the recombinant GSJ had good hydrolysis activity towards the terminal α-1, 4-glycosidic bond. With regard to the transglycosylation activity, the IMOs were synthesized by GSJ with a conversion rate of 37% when using maltose as a substrate. In industry, the yield of IMOs produced by α-glucosidase is generally reported to be about 60%. Although the IMO yield in our study was not ideal, optimizing the conditions of the transglycosylation reaction may raise the yields further. Additionally, because of its good thermal stability, the recombinant GSJ could be immobilized to recycle and reuse it, which could reduce the production costs.

## 4. Materials and Methods

### 4.1. Cloning and Expression of GSJ in *E. coli* BL21 (DE3)

The nucleotide sequence of a 1668-bp fragment encoding GSJ gene (AB154818) was synthesized by Synbio Technologies Inc. (Jiangsu, China). The synthesized GSJ was digested using *EcoR* I and *Xho* I, and then cloned into a pET-28a (+) vector with a strong T7 promoter, and the recombinant plasmid was transformed into the *E. coli* BL21 (DE3) for overexpression. A single colony of *E. coli* BL21 (DE3) harboring the recombinant plasmid was inoculated into a 10 mL Luria–Bertani (LB) culture-grown medium containing 0.1 mg/mL kanamycin and grown at 37 °C. The overnight culture was diluted into 50 mL terrific broth (TB), when the cell density was OD_600_ 0.5–0.6, the expression of the recombinant α-glucosidase was induced by 0.4 mM IPTG for continuous 36 h cultivation at 25 °C.

### 4.2. Assay of α-Glucosidase Activity

The hydrolysis activity of α-glucosidase was determined spectrophotometrically by measuring the amount of *p*-nitrophenol (*p*NP) released from *p*-nitrophenyl-α-glucopyranoside (*p*NPG, Sigma). The reaction mixture (200 μL) containing 10 mM *p*NPG, 15 mM phosphate buffer (pH 7.0) and appropriately diluted enzyme, was incubated at 65 °C for 10 min. The reaction was terminated by adding 50 μL of 1 M Na_2_CO_3_ and the liberated *p*NP was measured at 405 nm. One unit of α-glucosidase activity was defined as the amount of enzyme required to release 1 μmol *p*NP per minute under the conditions described above.

### 4.3. Enzyme Preparation and Purification

Cells were harvested and disrupted by sonication, and the intracellular soluble fraction was centrifuged at 12,000× *g* for 10 min to remove insoluble cell debris and proteins. The crude extracts of the enzymes were purified according to the following procedures. The intracellular soluble fraction was heated at 60 °C for 20 min and centrifuged at 12,000 for 10 min. The sample was then filtered with 0.22 μm filters (Jiangsu Green Union Science Instrument Co., Ltd.) followed by loading onto a Ni-NTA column. The fractions containing the target enzyme were pooled and dialyzed against phosphate buffer at 4 °C for 24 h. Proteins were monitored using sodium dodecyl sulfate-polyacrylamide gel electrophoresis (SDS-PAGE) and the Bradford method was used to determine the protein concentrations [26].

### 4.4. Effects of Temperature and pH on the Enzyme Activity and Stability

Temperature optimum and thermostability. The optimal temperature of α-glucosidase was determined by measuring the enzyme activity between 30 °C and 80 °C at pH 7.0, using *p*NPG as the substrate. The thermostability of α-glucosidase was determined by incubating the enzyme in 15 mM sodium phosphate buffer (pH 7.0) at 60 °C and 65 °C, and samples were assayed for residual activity at different intervals.

pH optimum and stability. pH optimum of α-glucosidase was measured at 65 °C over the range between 4.0–11.0 with increments of 1.0 pH unit, using 15 mM acetate buffer (pH 4.0–6.0), sodium phosphate buffer (pH 6.0–9.0) and glycine-NaOH buffer (pH 9.0–11.0). To determine the pH stability, the enzyme was incubated in the above various buffers at 4 °C for 24–48 h, followed by residual activity determination at pH 7.0 and 65 °C.

### 4.5. Metal Requirement

To determine the effect of metal ions on the recombinant α-glucosidase activity, the enzyme (30 ng) was preincubated with 1 mM of each metal ion (Ca^2+^, Mg^2+^, Fe^2+^, Zn^2+^, Mn^2+^, Ni^2+^, Cu^2+^ and K^+^) in 15 mM sodium phosphate buffer (pH 7.0) for 1 h at 65 °C. The residual enzyme activity was assayed at 65 °C.

### 4.6. Kinetic Parameters

Enzymatic assays for determining kinetic parameters were performed in the 15 mM sodium phosphate buffer (pH 7.0) at 65 °C with 0.1–4.0 mM *p*NPG as substrate. The apparent kinetic constants were calculated using the GraphPad software.

### 4.7. Substrate Specificity

The substrate specificity of purified GSJ was determined with various *p*NP derivatives (*p*NPαGlu, *p*NPβGlu, *p*NPαAra*p*, *p*NPαGal, *p*NPβGal, *p*NPαAra, *p*NPαRha, *p*NPβMan, *p*NPβXyl) as substrates. The reaction contained 1 mM *p*NP-glycoside, 15 mM sodium phosphate buffer (pH 7.0) and 2 μg recombinant enzymes were carried out at 65 °C for 10 min. All of the *p*NP derivatives were purchased from Sigma (St. Louis, MO, USA).

### 4.8. Hydrolysis Reaction

Commercial α-amylase (*Bacillus licheniformis*, A3403, Sigma) was added into the 1% (*w*/*v*) soluble starch and this mixture was incubated at 90 °C for 30 min. Then the mixture was incubated at 100 °C for 20 min to inactivate the enzyme, and centrifuged at 12,000× *g* for 15 min. After adding 5 ug recombinant GSJ or commercial α-glucosidase (*Bacillus. Stearothermophilus*, E-TSAGS, Megazyme, Ireland), the supernatant was incubated at 60°C. Following incubation for 1 h, the mixtures were incubated at 100 °C for 5 min to inactivate the enzyme, and centrifuged at 12,000× *g* for 15 min. The hydrolysis products were determined by HPAEC.

### 4.9. Transglycosylation Reaction

The reaction mixture, which consisted of 30% maltose and 5 μg enzyme in sodium phosphate buffer, was incubated at 60 °C for 2 h. Reaction mixtures were incubated at 100 °C for 5 min to inactivate the enzyme, and the samples were then centrifuged at 12,000× *g* for 10 min and analyzed by HPAEC.

### 4.10. HPAEC Analysis

Both the hydrolysis products and transglycosylation products were analyzed using HPAEC. The HPAEC-PAD analysis was performed on a Dionex ICS 5000 system with a CarboPac PA-200 column and pulsed electrochemical detector. The samples were eluted at 0.5 mL/min with a linear gradient from 0 to 50 mM NaAc in 50 mM NaOH for 20 min, and then eluted by 200 mM NaOH and 400 mM NaAc for 10 min. The standards of glucose, maltose, isomaltose, panose and isomaltotriose were obtained from Shanghai Hui Cheng Biological Technology Co. Ltd. (Shanghai, China).

### 4.11. MALDI-TOF Mass Spectrometry

The purified GSJ were dissolved into 0.1% trifluoroacetic acid (TFA). The reagent 2.5-dihydroxy benzoic acid was dissolved in 30/70 (*w*/*w*) acetonitrile/0.1% TFA as the matrix. Equal volumes of sample and matrix were mixed. The mixture (1 μL) was placed on an AnchorChip Standard (800 μm) plate and examined using a BRUKER Autoflex Speed mass spectrometer. Data were recorded using the FlexControl software and analyzed using FlexAnalysis software. Protein adduct ions [M + 2H]^2+^ or [M + H]^+^ were assigned.

## 5. Conclusions

In conclusion, α-glucosidase of the *Geobacillus* sp. strain HTA-462 was cloned and expressed in *E. coli*, and the recombinant enzyme was characterized in detail. The α-glucosidase activity in shake flasks was 107.9 U/mL. A detailed biochemical characterization showed that the recombinant α-glucosidase had high activity and stability at high temperatures and in a broad pH range. Additionally, the GSJ showed high α-1, 4-glucosidic hydrolysis and high transglycosylation activity, and converted maltose to IMOs with a yield of 37%. These studies indicated that the recombinant α-glucosidase has a great potential for industrial-scale applications. To the best of our knowledge, this is the first report to express the GSJ gene in *E. coli* and study IMO synthesis by the recombinant enzyme.

## Figures and Tables

**Table 1:** Purification scheme of recombinant GSJ.

Purification	Total Protein (mg)	Total Activity (U)	Specific Activity (U/mg)	Purification Fold	Yield (%)
Crude enzyme	90.90	3876.1	42.64	1.0	100.0
Heat treatment	25.05	3206.9	128.0	3.0	82.7
Ni-Sepharose	10.08	2856.0	283.3	6.6	73.7

**Table 2:** Kinetic parameter of recombinant GSJ.

Substrate	*K*m (mM)	*Vmax* (U mg^−1^)	*k*cat (s^−1^)	kcat/Km (mM^−1^s^−1^)
pNP-α-D-glucopyranoside	2.321	306.3	352	151.6

**Table 3:** Substrate specificity of recombinant GSJ on *p*NP derivatives.

Substrate	Relative Activity (%)
pNP-α-D-glucopyranoside	100.0
pNP-β-D-glucopyranoside	4.1
pNP-α-L-arabinofuranoside	6.6
pNP-α-L-arabinopyranoside	0.6
pNP-α-D-galactopyranoside	2.7
pNP-β-D-galactopyranoside	0.0
pNP-α-L-rhamnopyranoside	6.8
pNP-β-D-mannopyranoside	0.0
pNP-β-D-xylopyranoside	3.9
